# Antimicrobial resistance in community and nosocomial *Escherichia coli *urinary tract isolates, London 2005 – 2006

**DOI:** 10.1186/1476-0711-7-13

**Published:** 2008-06-18

**Authors:** David C Bean, Daniel Krahe, David W Wareham

**Affiliations:** 1 Centre for Infectious Disease, Institute of Cell and Molecular Science, Barts and The London, Queen Mary's School of Medicine and Dentistry, London, UK; 2 Department of Medical Microbiology, Homerton University Foundation NHS trust, London, UK; 3 Division of Infection, Barts and The London NHS Trust, London, UK

## Abstract

**Background:**

*Escherichia coli *is the commonest cause of community and nosocomial urinary tract infection (UTI). Antibiotic treatment is usually empirical relying on susceptibility data from local surveillance studies. We therefore set out to determine levels of resistance to 8 commonly used antimicrobial agents amongst all urinary isolates obtained over a 12 month period.

**Methods:**

Antimicrobial susceptibility to ampicillin, amoxicillin/clavulanate, cefalexin, ciprofloxacin, gentamicin, nitrofurantoin, trimethoprim and cefpodoxime was determined for 11,865 *E. coli *urinary isolates obtained from community and hospitalised patients in East London.

**Results:**

Nitrofurantoin was the most active agent (94% susceptible), followed by gentamicin and cefpodoxime. High rates of resistance to ampicillin (55%) and trimethoprim (40%), often in combination were observed in both sets of isolates. Although isolates exhibiting resistance to multiple drug classes were rare, resistance to cefpodoxime, indicative of Extended spectrum β-lactamase production, was observed in 5.7% of community and 21.6% of nosocomial isolates.

**Conclusion:**

With the exception of nitrofurantoin, resistance to agents commonly used as empirical oral treatments for UTI was extremely high. Levels of resistance to trimethoprim and ampicillin render them unsuitable for empirical use. Continued surveillance and investigation of other oral agents for treatment of UTI in the community is required.

## Background

*Escherichia coli *is the predominant cause of both community and nosocomial urinary tract infection (UTI). In the UK, trimethoprim or nitrofurantoin are usually recommended for empirical treatment of episodes of uncomplicated cystitis in the community [1], whilst parenteral cephalosporins and aminoglycosides are reserved for complicated infections or pyelonephritis. In North America a cut off point of 20% has been suggested as the level of resistance at which an agent should no longer be used empirically [2]. A UK study of the antimicrobial susceptibility of bacterial pathogens causing UTI in 1999 – 2000 showed high levels of resistance to trimethoprim, amoxicillin and oral cephalosporins [3] whilst a study of three collections of *E. coli *strains obtained from patients in East London in 1991, 1999 and 2004 showed rates of trimethoprim resistance of over 30% [4]. The emergence of strains producing extended spectrum β-lactamases (ESBL's) and others exhibiting quinolone resistance now threatens the empirical use of both cephalosporins and ciprofloxacin [5] seriously limiting treatment regimens. In order to determine current levels of resistance to antibiotics commonly used locally for empirical treatment, we reviewed susceptibility to ampicillin, amoxicillin/clavaulanate, trimethoprim, nitrofurantoin, cefalexin, gentamicin, ciprofloxacin and cefpodoxime amongst all *E. coli *urinary isolates obtained in our laboratory over a 1 year period.

## Methods

All *E. coli *isolates recovered from urine samples submitted for microscopy, culture and sensitivity to the laboratories of Barts and The London NHS Trust between 1^st ^January and 31^st ^December 2005 were included. Samples originating from General practice, Accident and Emergency or other primary care destinations were considered representative of community isolates whilst samples originating from patients hospitalised for 48 hrs or more on general or specialised wards were considered nosocomial.

Primary isolation of strains from urine specimens was performed using chromogenic agar (Mast diagnostics, Bootle, Merseyside) and bacterial counts quantified by inoculation of 0.3 μl of urine onto cystine lactose electrolyte deficient (CLED) agar (Mast diagnostics). Sensitivity testing was performed by the BSAC disc diffusion method using ampicillin (25 μg), cefalexin (30 μg), gentamicin (10 μg), ciprofloxacin (1 μg), nitrofurantoin (200 μg), trimethoprim (2.5 μg), amoxicillin/clavulanate (30 μg) and cefpodoxime (10 μg) discs and isosensitest agar.

Multi-drug resistance was defined in this analysis as resistance to three or more of the following antibiotics: ciprofloxacin, cefpodoxime, amoxicillin/clavulanate and gentamicin.

Differences in the prevalence of antibiotic resistance between groups were analysed using the χ^2 ^test. Strength of association was assessed by calculation of odds ratios with 95% confidence intervals.

## Results

A total of 11,865 *E. coli *isolates were cultured from urine samples over the study period, of these 10,521 (88.7%) were considered community isolates while 1,344 (11.3%) were of nosocomial origin. 10,166 (85.7%) were from women and 1,656 (14.0%) from men (43 sex unknown). 1,227 (10.3%) were from children < 16 years of age.

The frequency of antimicrobial susceptibility of all isolates to the eight antibiotics is shown in tables 1, 2, 3. Nitrofurantoin was the most active agent (94% susceptible) followed by gentamicin (93.7%) and cefpodoxime (92%). Ampicillin and trimethoprim were the least active agents with 55% and 40% of isolates exhibiting resistance respectively.

**Table 1:** Frequency of antibiotic susceptibility in relation to sex

Antibiotic	Female (*n *= 10157)		Male (*n *= 1656)		*P*	OR (CI95%)
				
	*n*	*n *(%) Resistant	*n*	*n *(%) Resistant		
Ampicillin	10153	5460 (53.8)	1652	1051 (63.6)	≤0.001	1.50 (1.35–1.67)
Amoxicillin/clavulanate	9178	1139 (12.4)	1491	310 (20.8)	≤0.001	1.85 (1.61–2.13)
Cefalexin	10139	892 (8.8)	1643	321 (19.5)	≤0.001	2.52 (2.19–2.90)
Ciprofloxacin	10137	1038 (10.2)	1649	374 (22.7)	≤0.001	2.57 (2.25–2.93)
Gentamicin	10149	525 (5.2)	1655	214 (12.9)	≤0.001	2.72 (2.30–3.22)
Nitrofurantoin	10134	551 (5.4)	1647	142 (8.6)	≤0.001	1.64 (1.35–1.99)
Trimethoprim	10138	3989 (39.3)	1652	748 (45.3)	≤0.001	1.28 (1.15–1.42)
Cefpodoxime	8512	525 (6.2)	1418	215 (15.2)	≤0.001	2.72 (2.29–3.22)

**Table 2:** Frequency of antibiotic susceptibility in relation to age

Antibiotic	< 16 years		≥ 16 years		*P*	OR (CI95%)
				
	*n*	*n *(%) Resistant	*n*	*n *(%) Resistant		
Ampicillin	1225	763 (62.3)	10484	5694 (54.3)	≤0.001	0.72 (0.64–0.81)
Amoxicillin/clavulanate	1109	143 (12.9)	9480	1296 (13.7)	NS	1.07 (0.89–1.29)
Cefalexin	1225	100 (8.2)	10462	1104 (10.6)	≤0.01	1.33 (1.07–1.64)
Ciprofloxacin	1224	72 (5.9)	10468	1334 (12.7)	≤0.001	2.34 (1.83–2.99)
Gentamicin	1226	44 (3.6)	10482	693 (6.6)	≤0.001	1.90 (1.39–2.59)
Nitrofurantoin	1224	46 (3.8)	10462	643 (6.1)	≤0.001	1.68 (1.24–2.28)
Trimethoprim	1223	566 (46.3)	10471	4129 (39.4)	≤0.001	0.76 (0.67–0.85)
Cefpodoxime	1064	43 (4.0)	8787	691 (7.9)	≤0.001	2.03 (1.48–2.78)

**Table 3:** Frequency of antibiotic susceptibility among community and nosocomial isolates

Antibiotic	Community		Nosocomial		*P*	OR (CI95%)
			
	*n*	*n *(%) Resistant	*n*	*n *(%) Resistant		
Ampicillin	10509	5663 (53.9)	1339	870 (65.0)	≤0.001	1.59 (1.41–1.79)
Amoxicillin/clavulanate	9564	1145 (12.0)	1145	307 (26.8)	≤0.001	2.69 (2.33–3.11)
Cefalexin	10498	876 (8.3)	1327	340 (25.6)	≤0.001	3.78 (3.29–4.36)
Ciprofloxacin	10488	974 (9.3)	1341	441 (32.9)	≤0.001	4.79 (4.20–5.46)
Gentamicin	10505	482 (4.6)	1342	260 (19.4)	≤0.001	5.00 (4.24–5.88)
Nitrofurantoin	10492	556 (5.3)	1332	139 (10.4)	≤0.001	2.08 (1.71–2.53)
Trimethoprim	10492	4103 (39.1)	1341	649 (48.4)	≤0.001	1.46 (1.30–1.64)
Cefpodoxime	8868	504 (5.7)	1103	238 (21.6)	≤0.001	4.57 (3.85–5.41)

Isolates from men were significantly more resistant to all eight agents than isolates from women (Table 1). In particular, resistance to cefpodoxime, gentamicin, ciprofloxacin and cefalexin was observed more than twice as frequently in isolates from men (odds ratios = 2.5). A significant difference between paediatric and adult isolates was seen for all agents except amoxicillin/clavulanate. Resistance to cefalexin, ciprofloxacin, gentamicin, nitrofurantoin and cefpodoxime was more common in adults whilst ampicillin (OR 0.72) and trimethoprim (OR 0.76) resistance was associated with paediatric strains (Table 2).

Nosocomial isolates were more resistant than community isolates to all agents tested. The prevalence of gentamicin (OR 4.93), ciprofloxacin (OR 4.74), and cefpodoxime (OR 4.48) resistance exhibited the most marked differences (Table 3). Patterns of multi-drug resistance are shown in table 4. Ampicillin resistance in combination with trimethoprim resistance was more frequently observed than resistance to the single agent alone, the combination of ampicillin and trimethoprim resistance was also seen in combination with amoxicillin/clavulanate and ciprofloxacin. Resistance to all agents except nitrofurantoin was the most common multi-drug resistant phenotype and was observed in 1.3% of isolates.

**Table 4:** Distribution of ten most frequently observed antibiotic resistance patterns.

Antibiotic	*n*	(%)
Susceptible	4290	36.13
AMP, TRI	2180	18.36
AMP	1835	15.46
TRI	691	5.82
AMP, AMC, TRI	292	2.46
AMP, AMC	265	2.23
AMP, CIP, TRI	241	2.03
AMP, AMC, LEX, CIP, GEN, TRI, CPD	164	1.38
AMP, AMC, LEX, CIP, TRI, CPD	98	0.83
AMP, NIT, TRI	95	0.80
Other	1722	14.50
*Total*	*10151*	*100*

AMP; ampicillin, TRI; trimethoprim, AMC; amoxicillin/clavulanate, CIP; ciprofloxacin, LEX; cefalexin, GEN; gentamicin, CPD; cefpodoxime, NIT; nitrofurantoin

## Discussion

In the UK most uncomplicated urinary tract infections are treated in the community with short courses of empirical antibiotics. This relies on susceptibility data from local surveillance schemes as in many cases urine samples are only sent for microbiological evaluation following treatment failure, recurrent or relapsing infection. Although the levels of resistance we observed amongst community isolates may therefore overestimate the true rate of resistance in the community, the high levels of resistance to ampicillin and trimethoprim raise concerns over the use of these agents. This was particularly evident amongst isolates from children, which were more likely to exhibit resistance to ampicillin and trimethoprim compared to those from adults. Increased resistance to the other agents in adults is likely to reflect their wider use both empirically and as second line therapies in relapsing, complicated or nosocomial infection. The higher rates of resistance to all agents observed in males are likely to reflect the complicated nature of UTI in men [6]. Infection in this group usually occurs in the setting of underlying anatomical or functional abnormalities or following instrumentation of the urinary tract and the use of prophylactic antimicrobials. Data on resistance rates in *E. coli *collected at another London teaching hospital from 1995 – 2000 reveal year on year increases in resistance to amoxicillin, cefuroxime, gentamicin and ciprofloxacin [7]. Resistance to comparable agents in 2005 shows marked elevations in resistance to gentamicin (6.3% v 3.2%) and in particular ciprofloxacin (12% v 1.9%). Resistance to cefpodoxime, which may signify ESBL production [8] was seen in 7.4% of isolates overall, often in combination with resistance to quinolones, aminoglycosides and trimethoprim. Although cefpodoxime resistance was more typical of nosocomial isolates, significant resistance was also observed in the community. These isolates most likely represent CTX-M producing strains of *E. coli *which have disseminated widely throughout Europe post 2000 [9] with those producing CTX-M-15 being most widespread in the UK [10].

## Conclusion

Nitrofurantoin remained the most active agent and as it can be administered orally and is highly concentrated in urine, it may therfore be the most appropriate agent for empirical use in uncomplicated UTI. Empirical treatment for nosocomial UTI or infection with multi-drug resistant isolates remains challenging with many authorities recommending parenteral carbapenems (imipenem, ertapenem or meropenem) [11] especially where ESBL producing isolates may be involved. The increasing rates of resistance to uropathogenic *E. coli *isolates reported worldwide [12,13] warrants evaluation of other treatments such as fosfomycin [14] or possibly novel cephalosporin/inhibitor combinations [15].

## Competing interests

The authors declare that they have no competing interests.

## Authors' contributions

All authors contributed equally to data extraction, analysis and drafting of the manuscript.
